# Treatment of Prosthetic Valve Thrombosis: Current Evidence and Future Directions

**DOI:** 10.14740/jocmr2392w

**Published:** 2015-10-23

**Authors:** Murat Biteker, Ibrahim Altun, Ozcan Basaran, Volkan Dogan, Birdal Yildirim, Gokhan Ergun

**Affiliations:** a Department of Cardiology, Faculty of Medicine, Mugla Sitki Kocman University, Mugla, Turkey; b Department of Emergency Medicine, Faculty of Medicine, Mugla Sitki Kocman University, Mugla, Turkey

**Keywords:** Prosthetic valve thrombosis, Treatment, Thrombolysis, Surgery

## Abstract

Prosthetic heart valve thrombosis (PVT) is a rare but serious complication with high morbidity and mortality. The optimal treatment of the PVT is controversial and depends on thrombus location and size, the patient’s functional class, the risk of surgery or thrombolysis, and the clinician’s experience. Although surgical therapy has been the traditional therapeutic approach, studies with low-dose and slow-infusion rates of thrombolytic agents have revealed excellent results. This article reviews the various treatment options in patient with PVT.

## Introduction

Rheumatic valve diseases are the most frequently encountered valvular pathology in developing countries and often require surgical replacement with prosthetic valves. However, prosthetic heart valve thrombosis (PVT) is a life-threatening complication whose management remains controversial [1]. PVT has an incidence from 0.1% to almost 6% per patient-year of left-sided valves and up to 20% of tricuspid valves [2]. The risk of PVT depends on valve type, anticoagulation status, valve position, the presence of prothrombotic states such as pregnancy, atrial fibrillation, and/or ventricular dysfunction. The most common cause of PVT is inadequate anticoagulant therapy [3]. PVT has been divided into two types, obstructive PVT (OPVT) and non-obstructive PVT (NOPVT). Although review of literature for management of PVT reveals no set guidelines, thrombolytic therapy, intensification of anticoagulation, thrombectomy, or prosthetic valve replacement are the currently available options [3]. In 1971, Luluaga et al were the first to use the thrombolytic therapy in PVT [4]. Streptokinase was used for treating thrombosis of thetricuspid valve prosthesis. Three years later, Baille et al reported the use of thrombolytic agent in a patient with aortic PVT [5]. Since then, several cases of PVT have been reported, with varied rates of success and complications.

There are a lack of randomized controlled prospective trials comparing surgical and thrombolytic therapies in PVT, but it is shown that intravenous slow infusion thrombolysis given in discrete, successive sessions guided by serial transesophageal echocardiography (TEE) can be achieved with a low risk of complications and a high rate of success even in patients with New York Heart Association (NYHA) class III or IV [6]. In this review, we will discuss the current treatment options in patients with PVT.

## Diagnosis of PVT

Clinical features remain important for the diagnosis of PVT in the modern era. OPVT can present along a wide spectrum that includes systemic embolism, fatigue, shortness of breath, acute haemodynamic deterioration and death [7]. Patients with NOPVT present minimal clinical symptoms and they are stable but they constitute a group of high embolic potential. Distinction between thrombus and pannus formation based on clinical grounds may be difficult. However, patients with thrombus formation have usually shorter duration of symptoms and more often inadequate anticoagulation. In the clinical suspicion of endocarditis, blood cultures should be performed. Although physical examination is frequently insufficient, it can reveal decreased prosthetic valve sounds, a new murmur, or change in a previously detected murmur.

The examination of a patient with prosthetic cardiac valve by transthoracic echocardiography (TTE) is an essential part of diagnostic assessment. TTE examination can be limited because the prosthesis produces a certain degree of acoustic shadowing and reverberations which need to be distinguished from vegetation or a thrombus. Doppler echocardiography is the most accurate method for detecting and quantifying the degree of transvalvar gradient increase and is useful in the follow-up of patients during thrombolysis. TEE can help to assess thrombus size and location by its high-resolution imaging and can aid in treatment decisions, such as thrombolysis, anticoagulation, and surgery. TEE along with clinical parameters can usually differentiate thrombus from pannus formation and vegetation. The thrombus size visualized by TEE is important in deciding on the optimal treatment strategy. When thrombolysis is contemplated, then TEE and Doppler echocardiography are the preferred modalities to assess serially the hemodynamic success of fibrinolysis. PRO-TEE study showed that, a thrombus area < 0.8 cm^2^ confers a lower risk for embolism or death associated with thrombolysis in left-sided OPVT [8].

Cinefluoroscopy provides the exact visualization of mechanical prosthetic heart valve leaflet motion [9]. It is readily available in most centers and can be performed rapidly, particularly in unstable patients. Fluoroscopy is not useful in distinguishing pannus from thrombus since neither pannus nor thrombus can be identified fluoroscopically. However, it is a low-cost, non-invasive imaging technique, with limited radiation exposure that allows the correct evaluation of opening and closing angles and the motion of the base ring of the prosthetic heart valve and can add diagnostic value to echocardiography [10]. It carries advantage over TEE for the visualization of leaflet motion in aortic prostheses, while the two modalities demonstrate comparable results in mitral prostheses.

Multidetector cardiac computed tomography (MDCT) is a promising technique for functional evaluation of bileaflet mechanical valves, allowing reliable measurements of opening and closing leaflet angles [11]. Although the exact cut-off attenuation values for the distinction between thrombus and pannus have not been established, MDCT may allow the differentiation of two entities, which is difficult with TEE mainly in the aortic position [12].

Real-time three-dimensional (3D) TEE provides a live “en face” surgical view of the valves, which can improve diagnostic accuracy for detecting prosthetic valve pathologies. The detection of NOPVT can be challenging, especially when Doppler parameters are within normal limits and clinical findings are subtle. Ozkan and colleagues found that real-time 3D TEE provides a more comprehensive delineation of non-obstructive mitral prosthetic valve ring thrombosis by depicting the morphology of thrombus with “en face” images that could be missed with 2D TEE [13].

## Treatment of PVT

**Figure 1:**
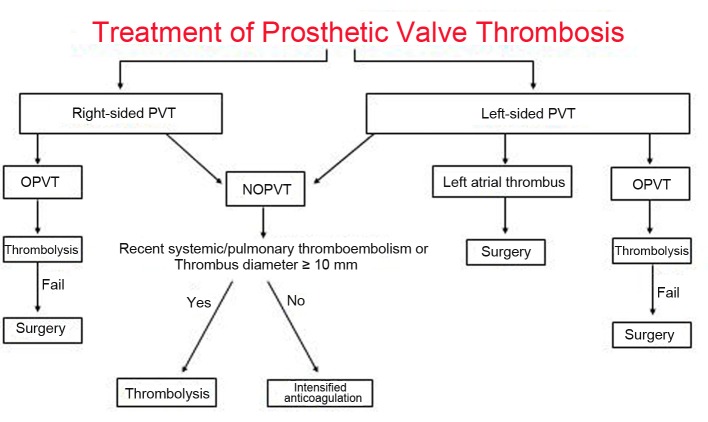
Treatment algorithm for patients with prosthetic valve thrombosis. OPVT: obstructive prosthetic valve thrombosis; NOPVT: non-obstructive prosthetic valve thrombosis.

The optimal managament of PVT remains controversial. The different therapeutic modalities available for PVT are largely influenced by the presence of valvular obstruction, by valve location (left- or right-sided), and by clinical status. In this review, we evaluated the management strategies of PVT according to presence of obstruction and prosthesis location (Fig. 1).

### Right-sided OPVT and NOPVT

PVT is the most important and common complication of the mechanical tricuspid valve. Mechanical prosthetic valves are rarely implanted in the right heart, mainly because of their important thrombogenicity. The incidence of mechanical tricuspid valve thrombosis may be up to 20% during the first postoperative year [14]. Although there are no formal prospective studies evaluating different treatment modalities, intensified anticoagulation should be the first choice of treatment in patients with non-obstructive right-sided PVT. Patients with obstructive tricuspid valve thrombosis usually present with signs of right heart failure, such as peripheral edema and ascites, and the prosthetic valve click may be inaudible during the auscultation. Both TTE and TEE can reveal the increased echogenicity on the prosthesis, decreased movement of the disc, and an elevated prosthetic valve gradient. The treatment of choice in right-sided OPVT is thrombolytic therapy, and fibrinolytic agents have been associated with a high success rate and a low complication rate [3]. There is no risk of cerebral embolism and the incidence of thromboembolism to the lungs is usually less serious than a cerebrovascular episode. Surgery should be reserved for cases with a pannus, thrombolytic failure, and contraindication to thrombolysis. Replacement of the mechanical tricuspid valve with a bioprosthesis can be considered in patients with failed thrombolysis, recurrent thrombosis, evidence of pannus or contraindications to thrombolytic therapy.

### Left-sided NOPVT

The management options of NOPVT are based mainly on small samples observational studies. The size of the thrombus is the most important factor determining the embolic potential of a NOPVT. When a cut-off value of 5 mm was used to define large thrombi, most complications occurred in patients where the NOPVT was > 5 mm in size. In a study conducted by Gueret and coworkers, all patients with a small (< 5 mm) NOPVT had an uneventful course with appropriate treatment, whereas five of the six patients with a large thrombus suffered a major embolic event [15]. Laplace et al, using the same cut-off of 5 mm, also reported similar results in a larger study [16], with early and late thromboembolic events numbering respectively one and three events in the group with a small thrombus (n = 29), and three and 11 events in the group with a large thrombus (n = 33). Moreover, while the embolic events were transient ischemic attacks in the small thrombus group, they were either stroke or clot enlargement causing obstruction of the valve in the large thrombus group.

Bemurat et al found that the prognosis is favorable with medical therapy by optimization of anticoagulant treatment (short-term intravenous unfractionated heparin followed by warfarin adjustment and aspirin addition) for small asymptomatic thrombi (length < 10 mm) [17]. If thrombus size is increased or is complicated by embolism, thrombolytic therapy or surgery should be considered [18]. The use of low-molecular-weight heparin in left-sided NOPVT is not clear yet.

According to the 2012 European Society of Cardiology and the European Association for Cardio-Thoracic Surgery guidelines, management of left-sided NOPVT depends mainly on the occurrence of a thromboembolic event and the size of the thrombus [19]. These guidelines recommend surgery for large (≥ 10 mm) NOPVT complicated by embolism (recommendation class IIa, level of evidence C) or which persists despite optimal anticoagulation [19]. Fibrinolysis may be considered if surgery is at high risk.

However, Nagy and coworkers reported that there was no significant difference in the outcome (successand complication) of thrombolytic treatment according to thrombus size [20]. These authors recommended thrombolytic treatment as the initial treatment in all PVTs, including all NOPVT, if the thrombus diameter is 5 mm or greater [20]. The TROIA study evaluated a strategy of TEE-guided fibrinolysis in with rapid infusion of streptokinase (group I) versus slow infusion of streptokinase (group II) versus full-dose tissue plasminogen activator (t-PA) (100 mg) (group III) versus half dose (50 mg) slow infusion of t-PA (group IV) versus low dose (25 mg) slow infusion of t-PA (group V) [6]. The investigators performed a single-center, prospective cohort study involving 182 patients with 220 episodes of PVT from 1993 to 2009, with a key feature of the study being that enrollment in the study arms was non-randomized and occurred sequentially during the study period. All patients with OPVT, patients with NOPVT with recent systemic thromboembolism, patients with asymptomatic NOPVT with a thrombus diameter of at least 10 mm, and PVT patients with ischemic stroke were included. Patients with asymptomatic NOPVT without a history of recent thromboembolism and with a thrombus diameter of < 10 mm were not included into the TROIA study. The investigators report successful thrombolysis in 83.2% of cases without a significant difference between thrombolytic protocols (68.8%, 85.4%, 75.0%, 81.5%, and 85.5%, respectively; P = 0.46). Analysis of complication rates by group showed a statistically lower combined complication rate in group V (10.5%) compared with all other groups (37.5%, 24.4%, 33.3%, 29.6%, and 10.5%, respectively; P = 0.01 for group I vs. group V, 0.03 for group II vs. group V, 0.04 for group III vs. group V, and 0.03 for group IV vs. group V). The PVT was obstructive in 105 episodes (47.7%) and non-obstructive in 115 (52.3%). Success rate was 87% in NOPVT, and 79% in OPVT (P = 0.12). Combined complication rates were 7.8% vs. 13.3% in NOPVT and OPVT groups (P = 0.18), respectively.

This study showed that the reduced-dose protocol (25 mg of tPA infused over 6 h) of thrombolytic treatment is effective with very low complications in patients with NOPVT and OPVT.

### Left-sided OPVT

The treatment of OPVT includes surgery (thrombectomy or valve replacement), thrombolytic therapy, and heparin; however, the optimal management is controversial. Once a diagnosis of prosthetic valve thrombotic obstruction has been made, heparin treatment should be started immediately. Unfortunately, heparin therapy is clearly inferior to both surgery and thrombolysis for obstructive thrombosis cases, and should not be considered a definitive treatment. Mortality rates following surgery mainly depend on the NYHA class of the patient; those patients in classes I to III have a mortality rate of 4.7%, whereas 60% of patients in class IV die during the intraoperative or postoperative period [21]. Roudaut and coworkers reported their non-randomized, retrospective, and single-center study on prosthetic valve obstruction in 210 patients (263 episodes) [22]. The study results showed that the two treatment arms had similar mortality rates (surgery 10% versus fibrinolysis 11%), and the authors favored surgical therapy over fibrinolysis as the embolic and major bleeding complications in the fibrinolytic group were higher than in those patients treated surgically (15% to 0.7%, and 4.7% to 0.7%, respectively). In addition, complete hemodynamic success was obtained in only 70% of cases with fibrinolytic therapy (compared to 89% with surgery). In an international multicenter registry (PRO-TEE study), patients with PVT underwent thrombolysis, and all of them had undergone TEE before therapy [8]. The registry comprised 107 patients, 93 of whom had OPVT, and 14 had NOPVT.

The agents used for fibrinolysis were streptokinase (54.7%), urokinase (17%), and t-PA (28.9%). All fibrinolytic agents were administered for a longer period of time, and streptokinase was even used for 120 h. The t-PA dosage was a 10-mg bolus, followed by 90 mg in 2 - 6 h. Complete hemodynamic success was achieved in 76.3% of the 93 obstructed valves and was similar among different valves and lytic agents. Partial hemodynamic success was infrequently seen (8.6%). This study found a previous history of cerebrovascular event and a thrombussize > 0.8 cm^2^ as one of the major risk factors for systemic embolic complications of thrombolytic therapy.

In the most recent European [19] and American guidelines [23], surgery is recommended for patients in NYHA functional classes III and IV unless surgery is high risk (class IIA). Thrombolysis is given a IIA indication in patients with right-sided valve thrombosis and a class IIB indication in patients with a left-sided but small thrombus. The European Society of Cardiology guidelines [24] also emphasize surgery for critically ill patients and restrict thrombolysis to patients with high surgical risk and/or right-sided valve thrombosis. However, the results of more recent studies have reported better outcomes with thrombolytic therapy than did the previous reports, and suggested that thrombolytic therapy would be the treatment of choice in all cases except for patients with contraindications to these agents. Caceres-Loriga and colleagues reported complete success in 85% of cases and partial success in 6% with thrombolytic therapy in their study with 68 patients during a 6-year period [24]. Nagy and coworkers reported the results of thrombolytic therapy in 62 OPVT cases [20]; complete success was achieved in 73% of these cases, and partial success in 21%, while the mortality (8%) and embolic complication (12%) rates of thrombolytic therapy were similar to those of previous studies, and superior to surgery.

In a recent literature survey 17 studies with clinical outcomes of 756 patients who received thrombolytic agents for treatment of 801 episodes of OPVT were analyzed [25]. Of the data that were available in 665 patients, 35% presented in NYHA functional classes I/II and 65% presented in NYHA functional classes III/IV. Complete success was achieved in 81% of patients presenting in NYHA functional classes I/II and 74% of patients presenting in NYHA functional classes III/IV. Streptokinase was used in 12 of the 17 studies. The rate of thromboembolism was 14% and the overall 30-day mortality was 8%.

In the largest series of patients with PVT, atrial fibrillation, obstructive thrombus, larger thrombus, and poor functional capacity, the so-called predictors of poor outcome in thrombolytic treatment of PVT, did not seem to predict the combined endpoint in PVT patients [6]. However, similar to the PRO-TEE study, thrombi > 0.9 cm^2^ were associated with increased major and minor embolic events. TROIA trial also showed that slow infusion of 25 mg t-PA without a bolus appears to be the safest thrombolytic regimen with lower complication and mortality rates for both OPVT and NOPVT compared with higher doses or rapid infusions of streptokinase or t-PA.

Patients with thrombotic material in the left atrium are at increased risk of major embolism and stroke when treated with thrombolytic therapy [26]. Although a few reports of successful thrombolysis of left atrial thrombi have been published [27], presence of a large left atrial thrombus is accepted as a contraindication for thrombolysis and should be ruled out by TEE before the start of thrombolytic treatment. However, there is no precise definition of the “large” thrombus in the current literature.

## Conclusions and Future Directions

One of the most life-threatening complications of mechanical prostheses is valvular obstruction by pannus, thrombus, or both. Until the 1990s, the treatment of choice for mechanical valve obstruction was surgery but over the last decade, thrombolyis has been used increasingly and has become an alternative to surgery as the first-line therapy in patients with PVT. Tissue plasminogen activator at a low dose and with prolonged infusion time has recently contributed to the success of thrombolytic therapy, with decreased complication rates. Further decrease of tPA with prolongation of the regimen may be associated with lower complication rates. Low-dose and ultra-slow infusion of tPA may be a preferred alternative treatment regimen for PVT in the future. The recently initiated two studies will provide important information for the management of PVT. SAFE-PVT (surgery versus fibrinolytic therapy for left-sided prosthetic heart valve thrombosis) study (NCT01641549) will randomize 150 patients at a single center in India to surgical valve replacement or thrombectomy versus first-line therapy with fibrinolysis with streptokinase or an alternative fibrinolytic agent. The second trial (NCT02243839) is a randomized and multicenter study, comparing thrombolytic therapy versus surgery for the treatment of patients with OPVT. Two different randomization groups are defined and patients with OPVT will be included in each group randomly. In the first arm, thrombolytic therapy will be performed to the patients. The thrombolytic therapy regimen depends on the functional status of the patient. In patients with NYHA class III-IV symptoms 25 mg tPA will be given in 6 h and in patients with NYHA class I-II dyspnea 25 mg tPA will be given in 25 h.
